# Supplementary material for: CDK8/19 inhibition attenuates G1 arrest induced by BCR-ABL antagonists and accelerates death of chronic myelogenous leukemia cells
Source: Cell Death Discov. 2025 Feb 15;11:62. doi: 10.1038/s41420-025-02339-6 (PMC11830074; doi:10.1038/s41420-025-02339-6)
Supplement: Supplementary file 1 — Supplementary file figures [file 41420_2025_2339_MOESM1_ESM.pdf]

K562

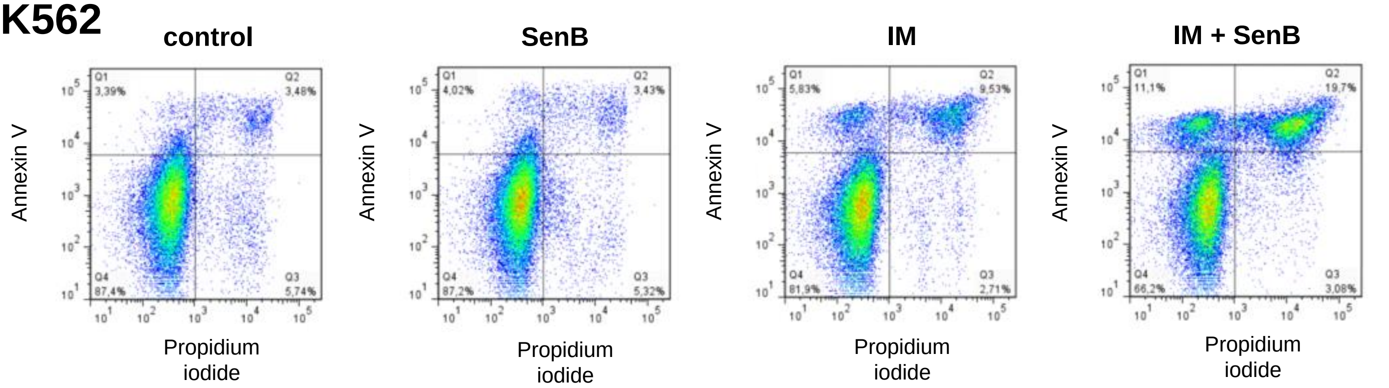

	control	SenB	IM	IM + SenB
Early apoptosis, Q1	3.39%	4.02%	**5.83%**	**11.10%**
Late apoptosis / necrosis , Q2	3.48%	3.43%	**9.53%**	**19.70%**
Necrosis, Q3	5.74%	5.32%	2.71%	3.08%
Live, Q4	87.4%	87.2%	81.90%	66.20%

KU812

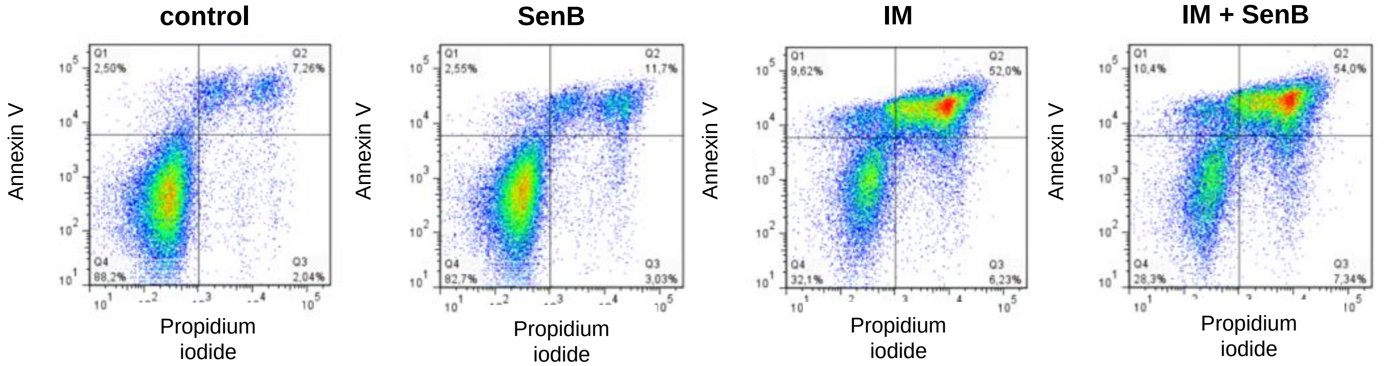

	control	SenB	IM	IM + SenB
Early apoptosis, Q1	2.50%	2.55%	**9.62%**	**10.40%**
Late apoptosis / necrosis , Q2	7.26%	11.70%	**52.00%**	**54.00%**
Necrosis, Q3	2.04%	3.03%	6.23%	7.34%
Live, Q4	88.20%	82.70%	32.10%	28.30%

Figure S1. Flow cytometry assisted analysis of death markers.

Upper panels: SenB in combination with IM trigger K562 cell death.
Bottom panels: No synergy between SenB and IM in KU812 cells.

Cells were treated with SenB, IM (1 μM each) or the combination for 24 h, washed and stained with Annexin V and PI (see *Materials and Methods* for details). Shown is one representative experiment out of three biological replicates.

Control vs. IM

Down	2100
NotSig	8174
Up	1756

Control vs. SenB

Down	679
NotSig	10334
Up	1017

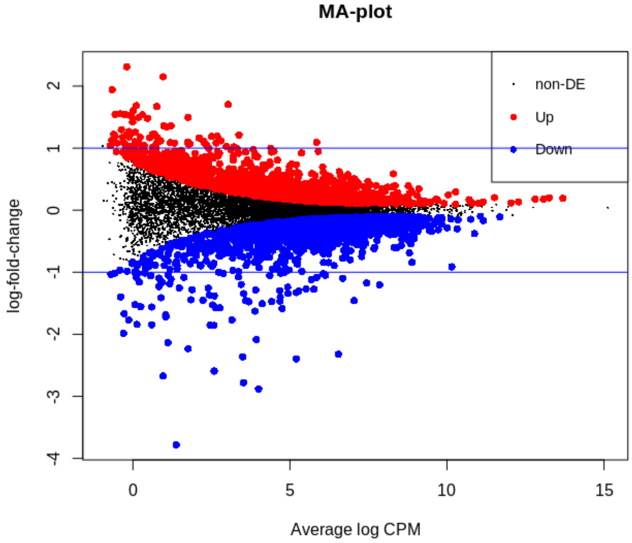

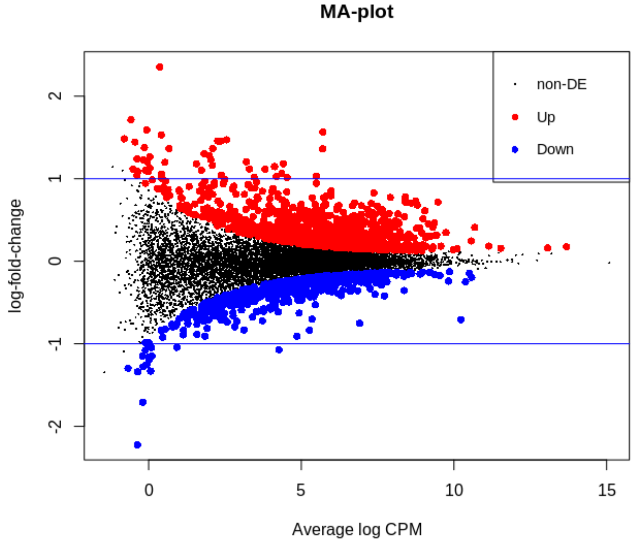

Figure S2. Down- and up-regulated genes in control vs. IM-treated K562 cells.

Figure S3. Down- and up-regulated genes in control vs. SenB-treated K562 cells.

Control vs. IM+SenB

Down	2394
NotSig	7563
Up	2073

IM vs. IM+SenB

Down	1185
NotSig	9353
Up	1462

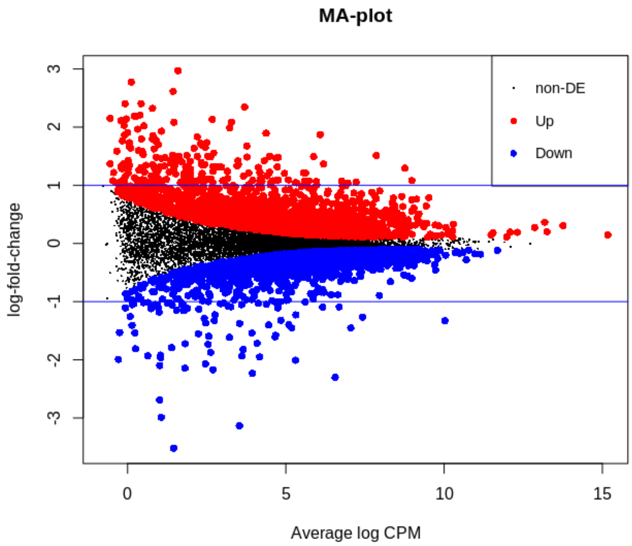

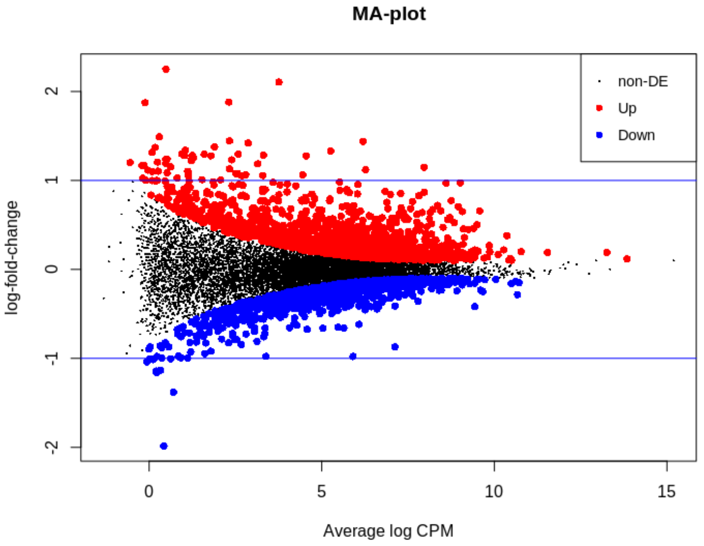

Figure S4. Down- and up-regulated genes in control vs. IM+SenB-treated K562 cells.

Figure S5. Down- and up-regulated genes in IM vs. IM+SenB-treated K562 cells.

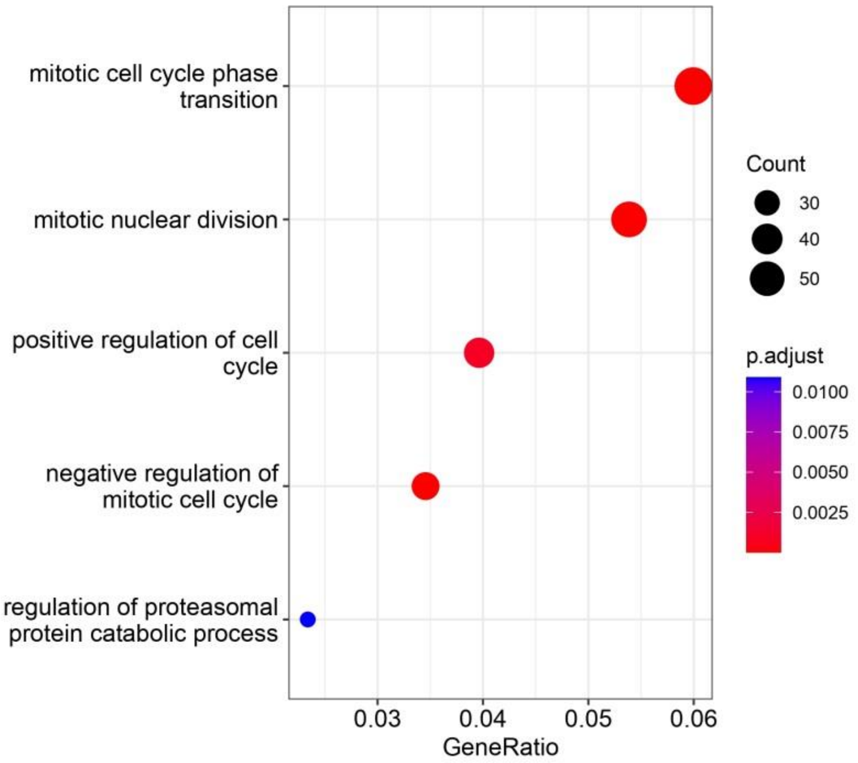

Figure S6. Down-regulated genes in IM vs. IM+SenB-treated K562 cells.

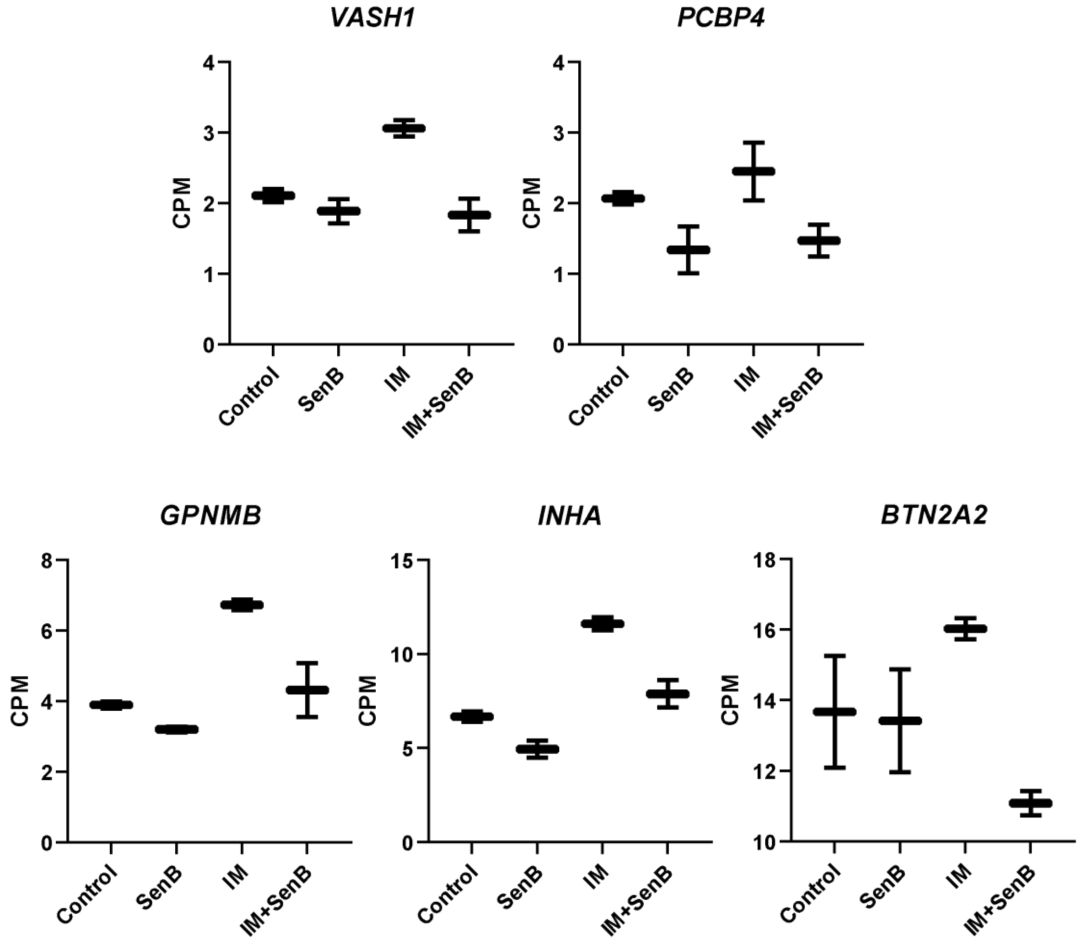

Figure S7. Top down-regulated cell cycle genes in IM vs. IM+SenB-treated K562 cells. Expression was measured by RNAseq.

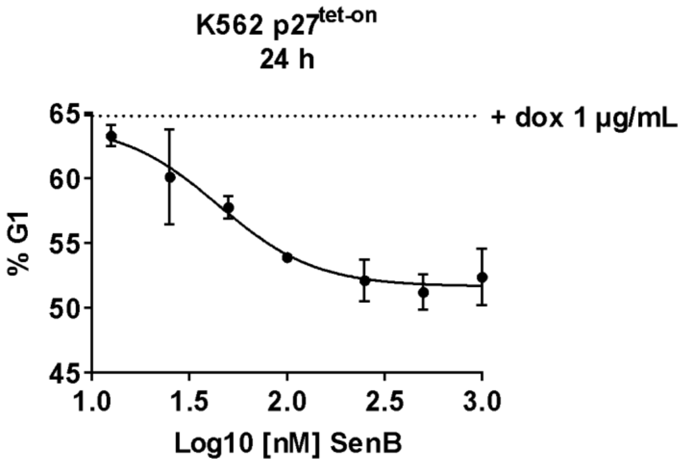

Figure S8. SenB attenuates G1 accumulation from doxycycline-induced p27^{Kip1}.
The K562p27tet-on cells were treated with 1 µg/ml doxycycline for 24 h in the absence or presence of 1 µM SenB. Cell cycle distribution was assessed by flow cytometry.

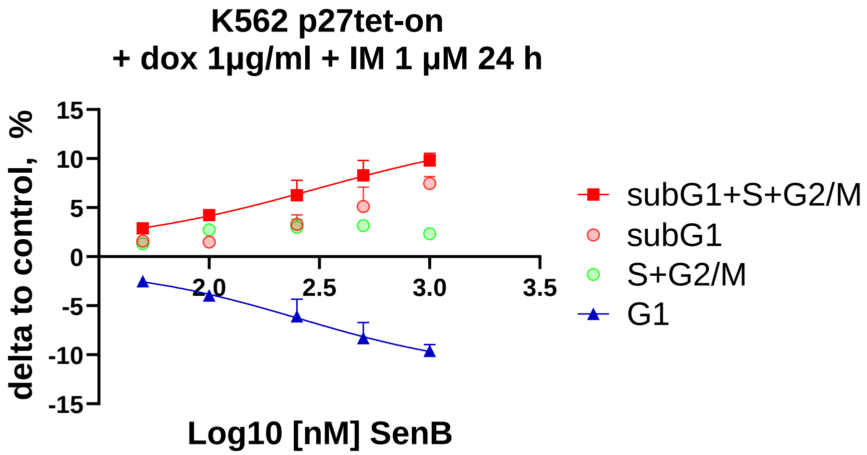

Figure S9. SenB in combination with IM contributes the decrease of G1 accumulation and increase of S+G2/M+subG1 fraction in doxycycline activated K562 p27tet-on cells.
The K562p27tet-on cells were treated with 1 µg/ml doxycycline for 24 h to induce p27$^{cip/kip}$ in the absence or presence of 1 µM SenB and in the presence of 1 µM IM. Cell cycle distribution was assessed by flow cytometry.

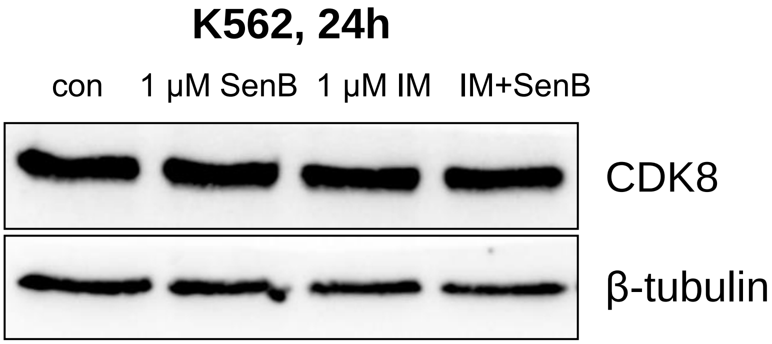

Figure S10. IM and SenB have no effect on CDK8 level in IM- and SenB-treated K562 cells. Level of CDK8 was analyzed by Western Blot and normalized to β-tubulin.
