# Supplementary material for: CDK8/19 inhibition attenuates G1 arrest induced by BCR-ABL antagonists and accelerates death of chronic myelogenous leukemia cells
Source: Cell Death Discov. 2025 Feb 15;11:62. doi: 10.1038/s41420-025-02339-6 (PMC11830074; doi:10.1038/s41420-025-02339-6)

Figure 1D

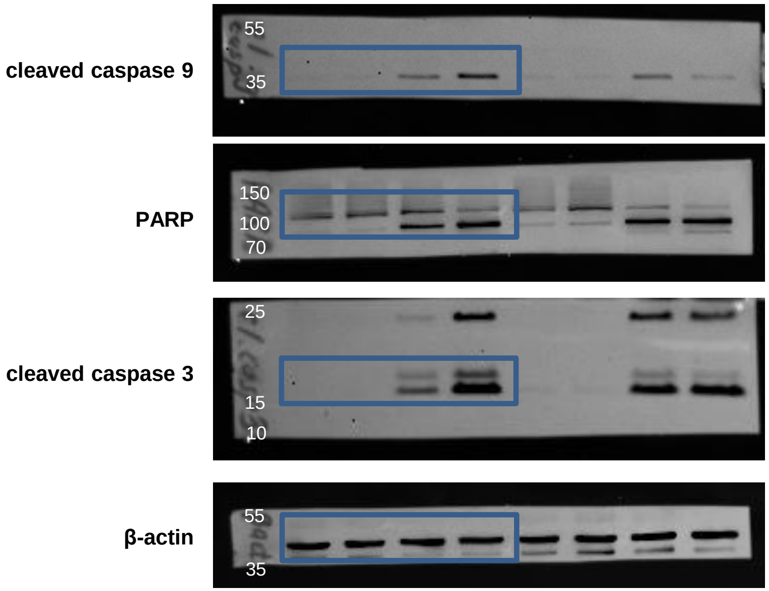

Figure 1E

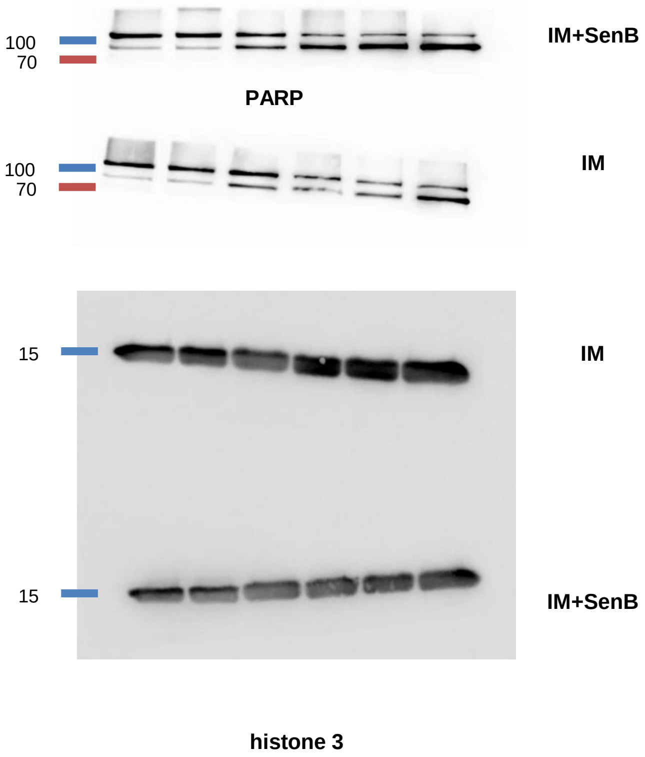

Figure 2B

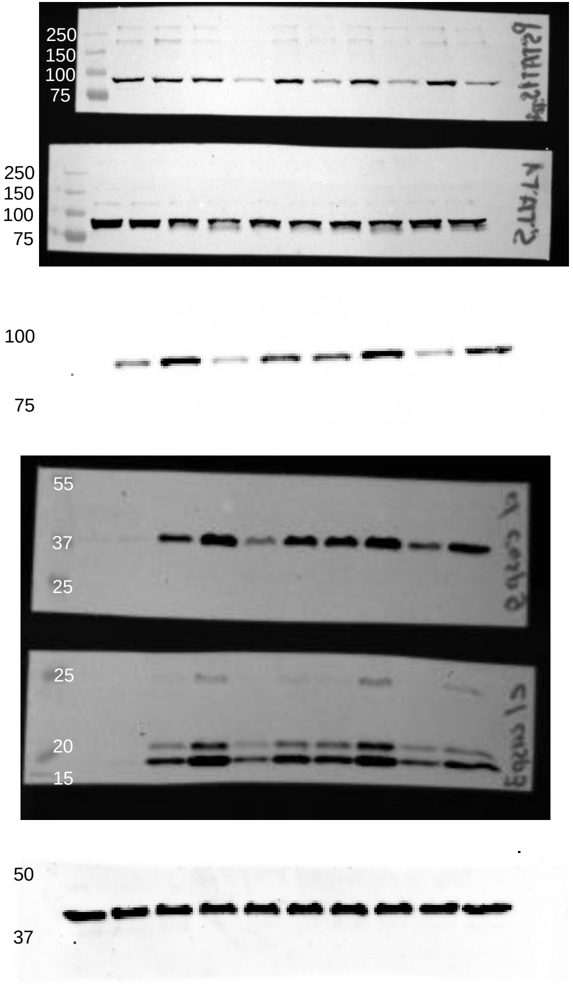

Figure 2D

pSTAT1 S727

STAT1

cleaved PARP

cleaved caspase 9

cleaved caspase 3

β-actin

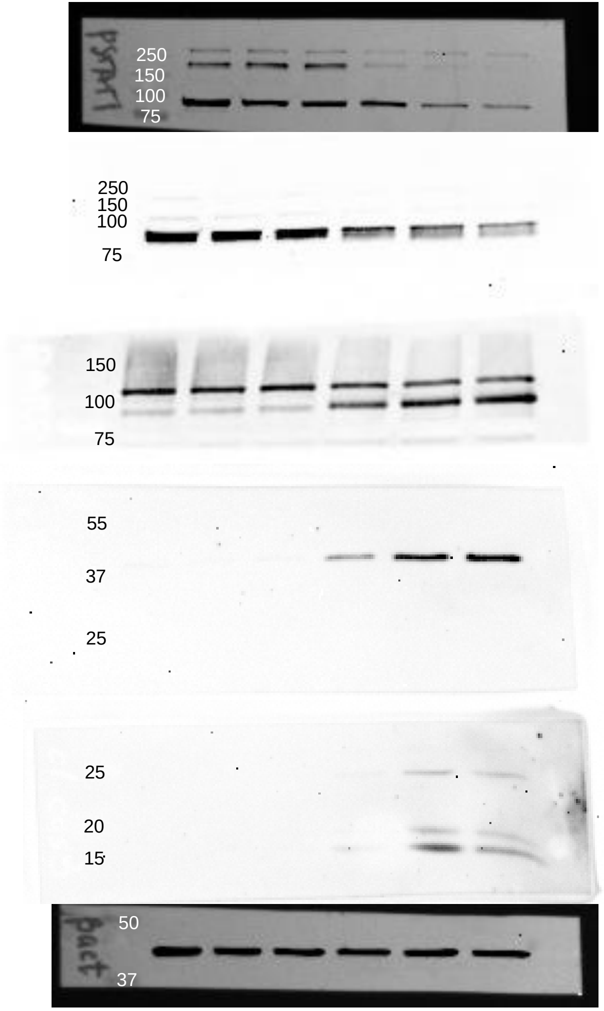

PARP

Figure 2E

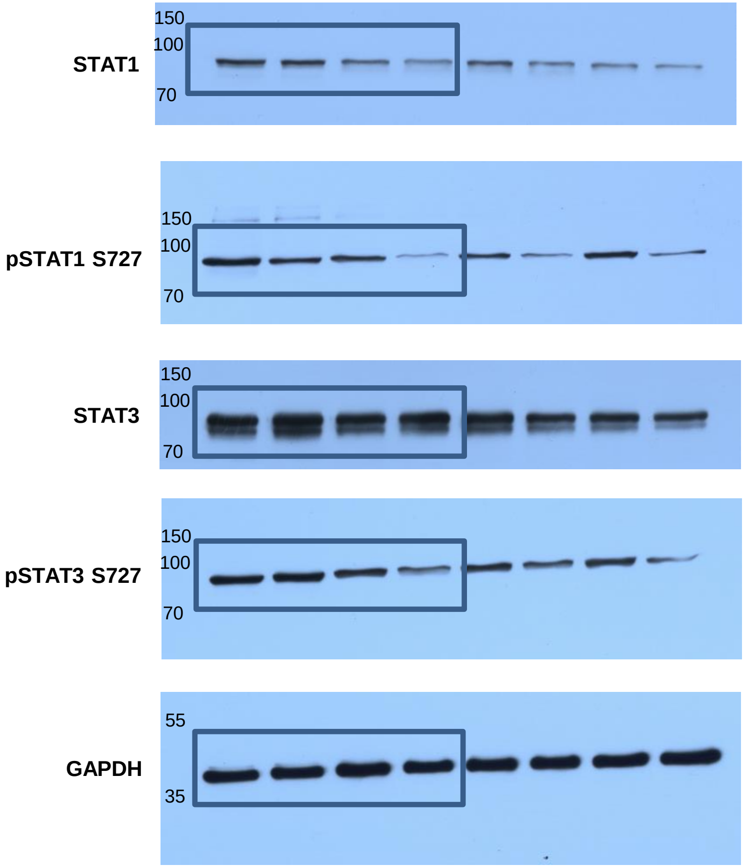

Figure 4B

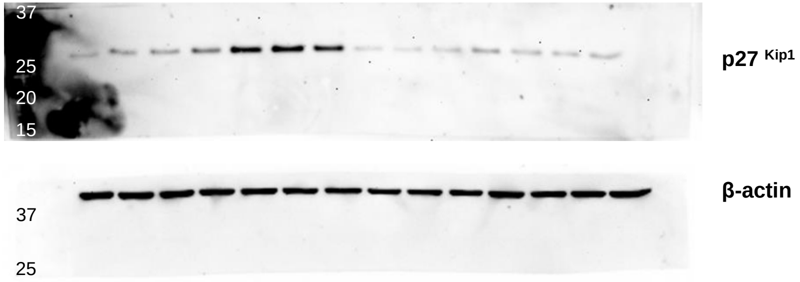

Figure 4C

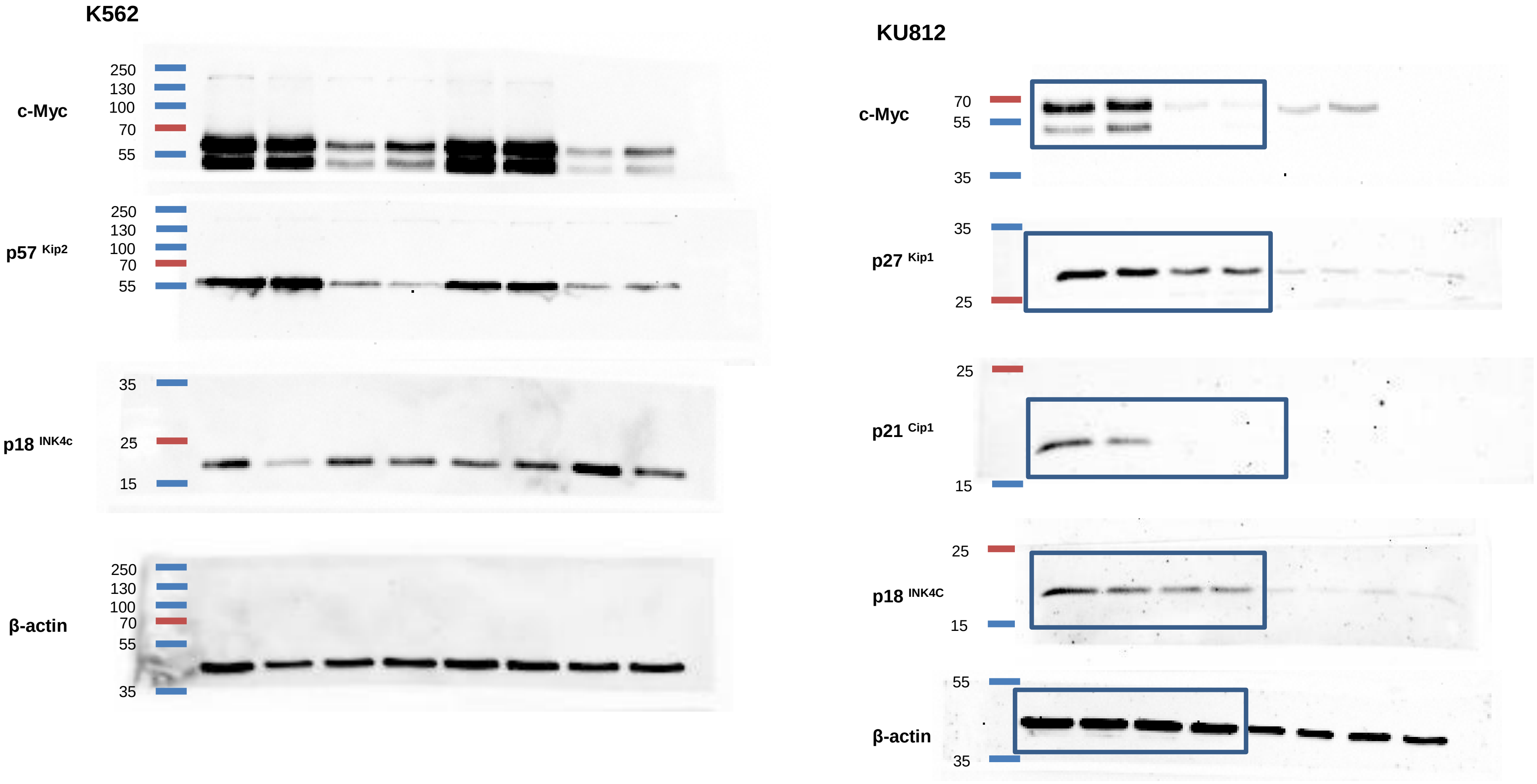

K562
c-Myc
250
130
100
70
55
p57 Kip2
250
130
100
70
55
p18 INK4c
35
25
15
β-actin
250
130
100
70
55
35
KU812
c-Myc
70
55
35
p27 Kip1
35
25
p21 Cip1
25
15
p18 INK4C
25
15
β-actin
55
35

Figure 5C

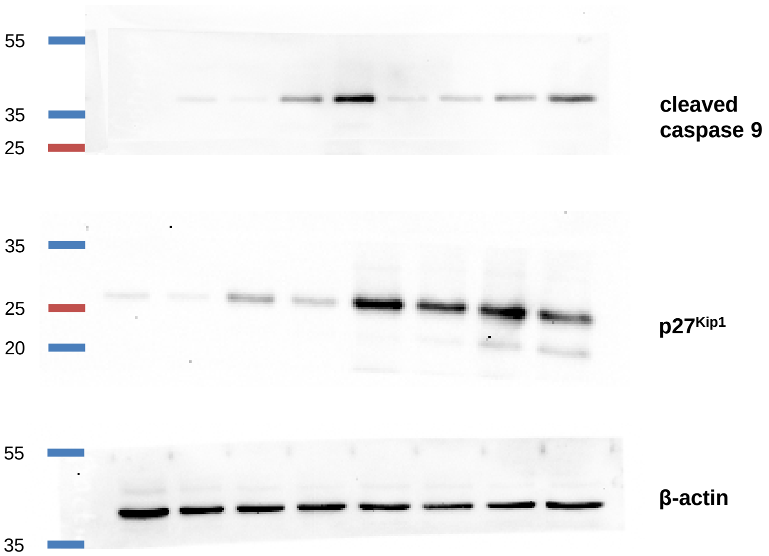

Figure 6C

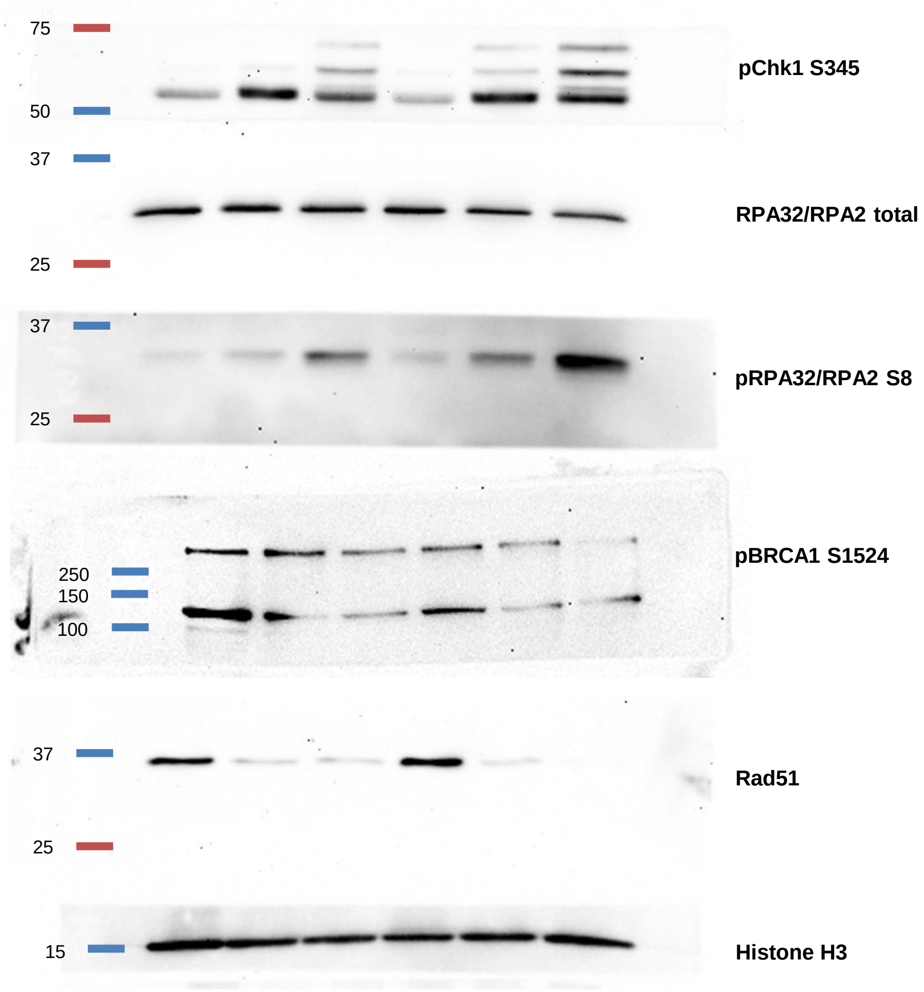

Supplement: Supplementary file 3 — Supplementary file WB [file 41420_2025_2339_MOESM3_ESM.pdf]
